# Low Dose Brain Radiation Reduces Elevated Plasma Branch Chain Amino Acid Levels in APP/PS1 Mice

**DOI:** 10.1093/geroni/igaa057.397

**Published:** 2020-12-16

**Authors:** Michael Maddens, George Wilson, Stewart Graham, Ali Yilmaz

**Affiliations:** 1 William Beaumont Hospital, Royal Oak, Michigan, United States; 2 Beaumont Hospital - Royal Oak, Royal Oak, Michigan, United States

## Abstract

Branch Chain Amino Acids (BCAA) have recently been implicated in Alzheimer’s Disease (AD). We previously showed that low dose brain radiation (RT) [5 fractions of 2 Gy] reduces amyloid-beta plaque burden and results in improved cognition in the APP/PS1 model of AD. In this study we investigated whether this schedule of radiation altered the metabolomic profile of serum. 10 month old male (M) and female (F) APP/PS1 mice were either treated with whole brain radiotherapy (5 x 2 Gy) or received sham irradiation. Eight weeks later the animals were euthanized and blood, urine and brain tissue collected. 1H NMR spectra were acquired. 256 transients were acquired for each sample and chemical shifts (δ) are reported in parts per million (ppm). Analysis included: 3 F and 5 M with no transgene (as a background controls), 5 F who received no RT, 7 F who received RT, 12 M who received RT and 12 M who received no RT. A total of 46 metabolites were analyzed. The most significantly changed metabolites were the BCAAs leucine, isoleucine and valine.. The effect was most pronounced in female mice where levels were reduced to those found in non-transgenic mice. APP/PS1 mice spontaneously display increased plasma BCAA, suggesting that AD pathology potentiates defects in BCAA metabolism, putting patients with AD at a higher risk of BCAA-induced brain damage. Reduction of these levels by low dose radiation may be beneficial.

